# Association Between Family Atmosphere and Internet Addiction Among Adolescents: The Mediating Role of Self-Esteem and Negative Emotions

**DOI:** 10.3389/ijph.2023.1605609

**Published:** 2023-06-26

**Authors:** Yijian Shi, Zijun Tang, Zhilin Gan, Manji Hu, Yang Liu

**Affiliations:** ^1^ Department of Psychology, Shanghai Normal University, Shanghai, China; ^2^ Shanghai Pudong New Area Mental Health Center, School of Medicine, Tongji University, Shanghai, China

**Keywords:** negative emotion, internet addiction, self-esteem, family atmosphere, chain mediation model

## Abstract

**Objectives:** Family atmosphere is a significant predictor of internet addiction in adolescents. Based on the vulnerability model of emotion and the compensatory internet use theory, this study examined whether self-esteem and negative emotions (anxiety, depression) mediated the relationship between family atmosphere and internet addiction in parallel and sequence.

**Methods:** A total of 3,065 Chinese middle school and high school students (1,524 females, mean age = 13.63 years, *SD* = 4.24) participated. They provided self-reported data on demographic variables, family atmosphere, self-esteem, anxiety, depression, and internet addiction through the Scale of Systemic Family Dynamic, Self-Esteem Scale, Self-Rating Anxiety Scale, Self-Rating Depression Scale, and Internet Addiction Test, respectively. We employed Hayes PROCESS macro for the SPSS program to scrutinize the suggested mediation model.

**Results:** It revealed that self-esteem, anxiety, and depression mediated the relationship between family atmosphere and internet addiction in parallel and sequence. The pathway of family atmosphere-self-esteem-internet addiction played a more important role than others.

**Conclusion:** The present study confirmed the mediating role of self-esteem and negative emotions between family atmosphere and internet addiction, providing intervention studies with important targeting factors.

## Introduction

Science and technology have made the internet more accessible to the general public. In particular, the virtual world has become an indispensable part of life for teenagers born during the internet age. According to China Minors’ Internet use Report 2020 [1], the internet penetration rate of minors in China reached 99.2%. Furthermore, the age of their first exposure to the internet declined over the year, with 78% having internet use experience before 10 years old. While the internet has brought some advantages to adolescents, such as attending online courses, the negative effects of excessive internet use should not be overlooked. Additionally, excessive internet use can lead to Internet Addiction Disorder (IAD) [2]. Internet addiction (IA) was coined by American psychiatrist Goldberg in 1994. It was characterized by continuous desires to surf the internet, out-of-control over internet use, withdrawal symptoms after removal, etc.

Scholars have made continuous attempts to investigate the social and physiological factors associated with IAD, and there is supporting evidence suggesting that family environment, negative emotions, and self-esteem play a significant role. Family atmosphere, or family environment, has long been recognized as an essential predictor of problematic internet use [3]. However, the pathways through which they are associated with each other remain unclear. In the meantime, negative emotions such as anxiety and depression have always been considered a result of improper family atmospheres [4] and a related issue to internet addiction among adolescents [5, 6]. Similarly, self-esteem is another potential mediator between family atmosphere and internet addiction among adolescents, as indicated by preceding literature [7, 8]. In brief, the present study explored the intermediate mechanisms that may rationalize the association between family atmosphere and internet use in adolescents by considering the role of negative emotions and self-esteem. The results may enlighten relevant intervention work.

### Family Atmosphere and Internet Addiction

The term “family atmosphere” pertains to the conditions and surroundings in which a child grows up. As a dimension of family characteristics, it was usually assessed by the Family Dynamic Scale and the Scale of Systemic Family Dynamic (SSFD) [9]. A study proposed that family atmosphere consists of three specific relational dimensions: the mother-child relationship, the parental relationship, and the parents’ restriction on children’s behavior [10].

Previous studies have indicated that the presence of an unsatisfactory family atmosphere, characterized by low levels of family satisfaction and cohesion and a high level of conflict between parents and children, is significantly associated with the development of internet addiction amongst adolescents [3, 11]. Moreover, longitudinal studies have shown that good family atmosphere can predict a substantial reduction of IAD in adolescents 2 years later [12], verifying their causal relationship. According to the compensatory internet use theory (CIUT [13]), adolescents who experience unsatisfactory family environments may turn to the internet as a means of escaping their reality and fulfilling unmet emotional needs, leading to the emergence of internet addiction [14]. This assumption has been confirmed by some preliminary studies conducted among Chinese middle school students [15]. Albeit these initial findings, the intermediate mechanisms that may account for the association of family atmosphere and internet use have not been examined empirically, amongst which are self-esteem and negative emotions (i.e., depression, anxiety).

### Self-Esteem as a Potential Mediator

Self-esteem is the positive or negative attitude towards oneself through social comparisons [16]. Accordingly, the main component of self-esteem is worthiness, such that high self-esteem implies that one thinks s/he is good enough [17]. The positive relationship between family atmosphere and self-esteem has been well-established among teenagers from different cultures [7, 18]. Meanwhile, self-esteem and problematic internet use were negatively associated, supported by both cross-sectional [19–25] and longitudinal study findings [26].

Researchers in the past have only indirectly verified the mediating role of self-esteem in the relationship between family atmosphere and internet addiction due to the use of various measures to assess family atmosphere. For instance, Yao et al. [22] found that self-esteem mediated parents’ warmth and internet addiction in Chinese adolescents. Similar findings were confirmed when family function was served as the independent variable [15]. The underlying mechanism may be that good family atmosphere promotes high self-esteem in adolescents, reducing their intention of seeking compensation on the internet and consequently decreasing the risks of internet addiction. According to Griffiths [27] and Niemz et al. [19], individuals with low self-esteem may derive great satisfaction from using the internet and treat it as a coping strategy to compensate for deficiencies, such as low self-esteem [19, 27].

### Anxiety and Depression as Potential Mediators

Negative emotions, such as anxiety and depression, typically refer to extreme emotional reactions and persistent maladaptive emotions that negatively evaluate one’s situation [28, 29]. Though anxiety and depression often co-occur, they are independent of each other and represent distinguishable categories of negative emotions [30].

Adolescents with unhealthy family atmosphere are likely to experience anxiety and depression. For instance, Berryhill and Smith [31] found a positive relationship between chaotically disengaged family functioning and anxiety symptoms. Similarly, poor systematic family dynamics, lack of intimacy and disharmony were predictors of depression and suicidal ideas in adolescents [32–36].

Adolescents’ negative emotions were proven to predict internet addiction over time. For example, high anxiety [37] and depression [38] were risk factors of problematic internet use [39]. According to the compensatory internet use theory, adolescents may use the internet to alleviate these negative emotions [13].

To sum up, adolescents may attempt to alleviate anxiety and depression caused by negative family environments by turning to the internet, a topic that necessitates empirical examination.

### Chain Mediation of Self-Esteem and Negative Emotions

The two sources of mediators proposed above are also closely associated with each other [40]. Specifically, individuals with low self-esteem were more susceptible to negative emotions, potentially because being in a state of low self-esteem for a long time weakened their ability to regulate emotions [41–43]. This is in line with the vulnerability model of emotion, suggesting low self-esteem increases the likelihood of experiencing depression and anxiety (see meta-analysis [44]).

Though the two negative emotions of concern often co-occur in adolescents [45], anxiety was found more robustly predicted depression than the other way around [46, 47]. The potential reason is that anxiety creates a sense of helplessness [48], which in turn precipitates depressive signs of despair and perceived loss [49]. In sum, family atmosphere may be associated with internet addiction via four chain mediation pathways: self-esteem—anxiety, self-esteem—depression, self-esteem—anxiety—depression, and anxiety—depression.

### The Present Study

In sum, there is a lack of literature investigating the mediating role of self-esteem and negative emotions between family atmosphere and problematic internet use. The present study aims to fill this research gap by conducting a relatively large-scale survey among Chinese adolescents. Furthermore, in line with previous studies, factors that may confound the examination of the proposed model were controlled, including age, gender, and self-rated family financial status [24, 50]. We hypothesized that: 1) good family atmosphere is negatively associated with internet addiction; 2) self-esteem and negative emotions partially mediate (1) in parallel and sequence.

## Methods

### Participant

We employed a multi-stage stratified sampling technique to obtain our data. Initially, we randomly chose two districts out of the 16 districts situated in Shanghai. Subsequently, we randomly selected three middle and three high schools from each district. Finally, we randomly picked two classes from every grade level of each school. Participants filled out the paper-pencil test under the guidance of their psychological teachers in school, and 3,358 complete copies were received (response rate of 81.7%). The answers of 293 students were discarded due to high missing data rates, resulting in 3,065 participants (1,527 girls, 13.63 ± 4.24 years) included in the data analyses. Approval from the school principals was obtained before the survey, and written consent was obtained from all participants and their parents.

### Instruments

#### Sociodemographic Form

Sociodemographic information was collected, including age, gender, grade, and self-rated family financial satisfaction (0: *not at all satisfied* to 10: *completely satisfied*).

#### Family Atmosphere

Family atmosphere was measured by the Self-rating Scale of Systemic Family Dynamic (SSFD [51]), which contains 23 items and four dimensions (i.e., family atmosphere, individualization, system logic, and illness concept). Adolescents rated items on a 5-point Likert scale from 1 (*completely disagree*) to 5 (*completely agree*). The eight items that belong to family atmosphere dimension were included in the analysis. A higher score indicates a better family atmosphere. The validated Chinese version was used [52], and Cronbach’s alpha of the family atmosphere dimension was 0.87 in the present study.

#### Self-Esteem

Self-esteem was measured by the Self-Esteem Scale (SES) developed by Rosenberg [17]. It consists of 10 items, rated on a 4-point Likert scale from 1 (*completely disagree*) to 4 (*completely agree*). After reversely scoring items 3, 5, 9, and 10 and adding them up with the rest, a higher SES total score indicates high self-esteem. The Cronbach’s alpha coefficient of SES was good (0.89) in the present study**.**

#### Anxiety

Anxiety was measured by the Self-Rating Anxiety Scale (SAS) developed by Zung ([53]). It consists of 20 items, rated on a 4-point Likert scale ranging from 1 (*no or little time*) to 4 (*most or all of the time*). A total SAS score was calculated by first reversely scoring items of 5, 9, 13, 17, and 19, then adding up each item and multiplying it by 1.25. A score below 50, between 50 and 59, between 60 and 69, and above 70 indicates free from anxiety, mild anxiety, moderate anxiety, and severe anxiety, respectively. The Cronbach’s alpha coefficient of SAS was good (0.82) in the present study.

#### Depression

Depression was measured by the Self-Rating Depression Scale (SDS) developed by Zung [54]. It consists of 20 items, rated on a 4-point scale from 1 (*no or little time*) to 4 (*most or all of the time*). A total SDS score was calculated by first reversely scoring items of 2, 5, 6, 11, 12, 14, 16, 17, 18, and 20, then adding up each item and multiplying it by 1.25. A score below 53, between 53 and 62, between 63 and 72, and above 73 indicates free from depression, mild depression, moderate depression, and severe depression, respectively. The Cronbach’s alpha coefficient of SDS was good (0.84) in the present study.

#### Internet Addiction

Internet addiction was measured by the Internet Addiction Test (IAT-20) developed by Young [55]. The questionnaire comprises 20 items, rated on a 5-point Likert scale from 1 (*not at all true*) to 4 (*always true*), with higher total scores representing higher degrees of internet addiction. The validated Chinese version was used in the present study, which has good reliability and validity among Chinese adolescents [56]. The Cronbach’s alpha coefficient of IAT was excellent (0.90) in the present study.

### Analytic Plan

We examined the missing data rate for all variables and used mean score imputation for variables with less than 10% missing data [57]. Two demographic variables, namely, parent’s education level and the number of siblings, were excluded from the analyses because they contained over 10% missing data. Afterward, descriptive statistics and correlations between variables were calculated. Lastly, Hayes’ PROCESS macro, version 4.0, model 6 (a linear model) [58] was used to test the chain mediation model through which family atmosphere was hypothesized to influence internet use. Mediators examined included self-esteem, anxiety, and depression. In addition, demographic variables (i.e., age, gender, and self-rated family financial satisfaction) that may confound the mediation model were controlled by adding them as covariates. Bootstrapping procedures with 5,000 replications were used to calculate confidence intervals for estimates of the indirect effects, with the confidence interval did not include zero indicating a significant indirect effect. Pairwise contrasts compared the relative strength of the indirect pathways involved in a mediation model. Furthermore, the effect sizes of the mediation effects were represented by the proportion of the total effect explained by the indirect effect [59].

## Result

### Participant Characteristics

In the present study, the average score of family atmosphere, self-esteem, anxiety, depression, internet addiction, and self-rated family financial satisfaction was 65.46 (*SD* = 22.59), 30.84 (*SD* = 6.11), 41.83 (*SD* = 33.59), 46.49 (*SD* = 29.88), 41.75 (*SD* = 11.31), and 7.82 (*SD* = 1.98), respectively. As was shown in Table 1, 50.3%, 19.9%, and 28.1% of the participants had experienced internet addiction, anxiety, and depressive symptoms, respectively. Furthermore, the detection rate of anxiety and depression was higher in females than in males (anxiety: *χ* ^2^ = 31.97, depression: *χ* ^2^ = 8.55), thus gender was controlled in the mediation analysis.

**TABLE 1:** The detection rate and gender difference of internet addiction, anxiety, and depression in adolescents (China, 2022).

Variables		Internet addiction [*n* (%)]		*χ* ^*2*^	*p*	Anxiety [*n* (%)]		*χ* ^*2*^	*p*	Depression [*n* (%)]		*χ* ^*2*^	*p*
		No	Yes			No	Yes			No	Yes		
Gender	Male	773 (48.5)	778 (51.5)	0.159	0.69	1,308 (84.3)	243 (15.7)	31.966	0.00	1,156 (74.5)	395 (25.5)	8.548	0.03
	Female	772 (50.6)	755 (49.4)			1,164 (76.2)	363 (23.8)			1,066 (69.8)	461 (30.2)		
Total		1,524 (49.7)	1,541 (50.3)			2,456 (80.1)	609 (19.9)			2,203 (71.9)	862 (28.1)		

### Correlation Analyses Between Main Variables

As expected, family atmosphere was positively associated with self-esteem (*r* = 0.522, *p* < 0.01) and negatively associated with internet addiction (*r* = −0.37, *p* < 0.01), anxiety (*r* = −0.457, *p* < 0.01) and depression (*r* = −0.551, *p* < 0.01) (Table 2). Internet addiction was negatively associated with self-esteem (*r* = −0.393, *p* < 0.01) and positively associated with anxiety (*r* = 0.402, *p* < 0.01) and depression (*r* = 0.405, *p* < 0.01). In addition, age and family financial satisfaction was significantly associated with all the examined variables (Table 2), and thus were controlled in the mediation model.

**TABLE 2:** Correlations between family atmosphere, internet addiction, self-esteem, anxiety, depression, age, and family financial satisfaction (*n* = 3,065) (China, 2022).

Variables	M	SD	(1)	(2)	(3)	(4)	(5)	(6)
FA	65.46	22.59						
IAT	41.75	14.40	−0.37**					
SES	30.84	6.10	0.52**	−0.39**				
SAS	41.84	10.52	−0.46**	0.40**	−0.55**			
SDS	46.49	11.85	−0.55**	0.41**	−0.69**	0.75**		
Age	13.63	2.01	−0.23**	0.31**	−0.09**	0.09**	0.09**	
Family financial satisfaction	7.82	1.98	0.40**	−0.27**	0.32**	−0.28**	−0.30**	−0.17**

Note. **p < 0.01. FA, family atmosphere; SES, self-esteem; SAS, anxiety; SDS, depression; IAT, internet addiction.

### Chain Mediation Model

**FIGURE 1:**
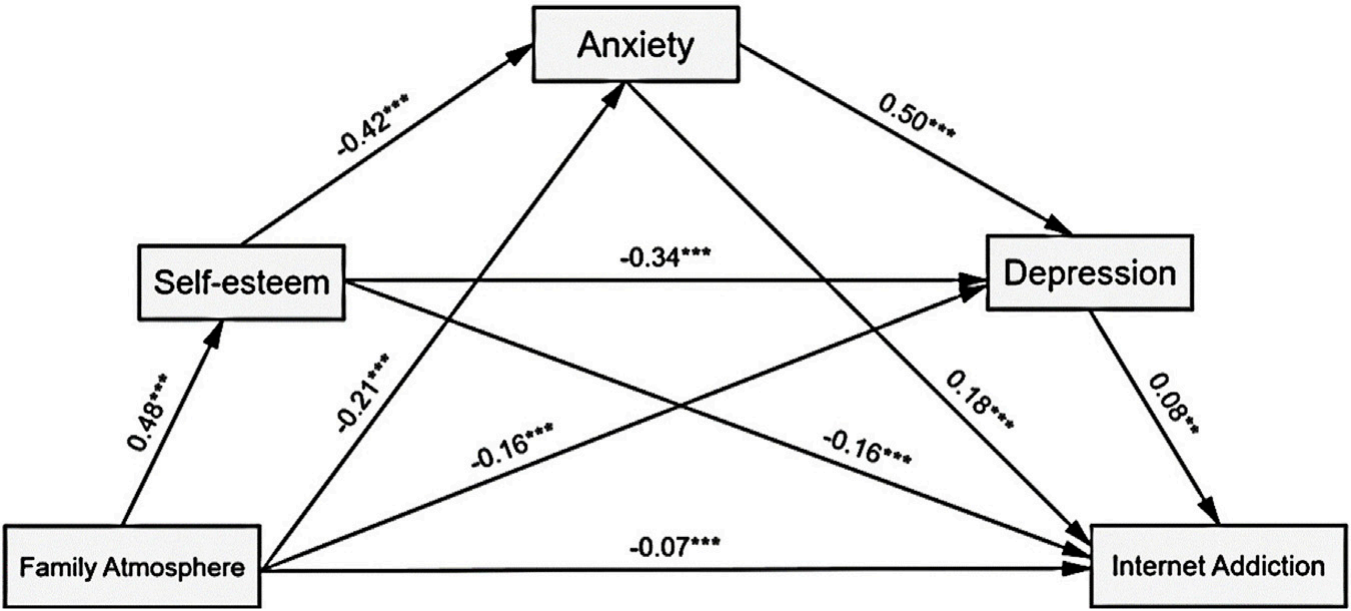
The chain mediation model of the association between family atmosphere and internet addiction with self-esteem, anxiety, and depression as mediators. The path estimation shown with each arrow is the standardized coefficient after adjusting for age, gender and self-rated family financial satisfaction. ***p < 0.001, **0.001 < *p* < 0.01 (China, 2022).

The overall model accounted for 53.5% of the variance in the internet addiction total score (*R* ^2^ = 0.29, *F* (7, 3,029) = 173.28, *p* < 0.001). As presented in Figure 1, family atmosphere had a significant positive predictive effect on self-esteem (*β* = 0.48, *p* < 0.001), and it negatively predicted anxiety (*β* = −0.21, *p* < 0.001), depression (*β* = −0.16, *p* < 0.001), and internet addiction (*β* = −0.07, *p* < 0.001). Furthermore, self-esteem negatively predicted anxiety (*β* = −0.42, *p* < 0.001), depression (*β* = −0.34, *p* < 0.001), and internet addiction (*β* = −0.16, *p* < 0.001). Meanwhile, anxiety had a positive relationship with depression (*β* = 0.50, *p* < 0.001) and internet addiction (*β* = 0.18, *p* < 0.001), and depression also positively predicted internet addiction (*β* = 0.08, *p* < 0.01).

The direct effect of family atmosphere on internet addiction was significant (*β* = −0.0580, SE = 0.0124, 95% CI = [−0.0824, −0.0336]), as were the seven indirect pathways (Figure 1; Table 3). Roughly 73.9% of the variance in internet addiction accounted for by family atmosphere was explained by self-esteem (PM = 29.1%), anxiety (PM = 14.8%), depression (PM = 4.8%), self-esteem-anxiety (PM = 13.8%), self-esteem-depression (PM = 5.0%), anxiety-depression (PM = 3.3%), and self-esteem-anxiety-depression (PM = 3.1%). In particular, the indirect effect of “family atmosphere—self-esteem—internet addiction” was considerably more significant than the other six indirect pathways (Table 3). Two important secondary pathways were family atmosphere-anxiety-internet addiction, and family atmosphere-self-esteem-anxiety-internet addiction.

**TABLE 3:** The model for the mediating effect of self-esteem, anxiety, and depression on the association of family atmosphere and internet use (*n* = 3,065) (China, 2022).

Paths	*β*	SE	95% CI		*P*_*M*_	Relative strength of the indirect effect
			Lower	Upper		
1. FA-SES-IAT	−0.076	0.011	−0.099	−0.053	29.1	>2, 3, 4, 5, 6, 7
2. FA-SAS-IAT	−0.038	0.007	−0.053	−0.026	14.8	>3, 5, 6, 7
3. FA-SDS-IAT	−0.012	0.005	−0.022	−0.003	4.8	>6, 7
4. FA-SES-SAS-IAT	−0.036	0.006	−0.048	−0.025	13.8	>5, 6, 7
5. FA-SES-SDS-IAT	−0.013	0.005	−0.023	−0.003	5.0	>6, 7
6. FA-SAS-SDS-IAT	−0.009	0.003	−0.016	−0.002	3.3	
7. FA-SES-SAS-SDS-IAT	−0.008	0.003	−0.014	−0.002	3.1	

Note. Values reported are standardized effects. FA, family atmosphere; SES, self-esteem; SAS, anxiety; SDS, depression; IAT, internet addiction; P_M_ refers to the proportion of the total effect explained by the indirect effect. Relative strength of the indirect effect is calculated by pairwise contracts of standardized indirect coefficients.

## Discussion

The present study examined the mediating chain of self-esteem—anxiety—depression in the relationship between family atmosphere and internet addiction within a large sample of Chinese adolescents. We found that family atmosphere was positively associated with self-esteem, and negatively associated with internet addiction, anxiety and depression. Secondly, self-esteem, anxiety, and depression mediated the relationship between family atmosphere and internet use in parallel and sequence, which was in line with our hypotheses. Next, we start with explanations and implications of the main findings, followed by limitations and suggestions for future studies.

### Family Atmosphere and Internet Addiction

Consistent with previous studies, we found that inferior family atmosphere increased the risk of adolescents’ internet addiction [60, 61]. As one of the earliest and most intimate growing environments [62], family atmosphere profoundly affects adolescents’ mental health [63]. According to the compensatory internet use theory [13], adolescents exposed to long-term familial stress and conflict are likely to use the internet to meet their unmet needs (e.g., satisfaction and pleasure [64, 65]). In contrast, warm and harmonious family atmosphere provides adolescents with a sense of security and belonging, which help meet their psychological needs and protect them from internet addiction [66].

### The Mediating Role of Self-Esteem

As one of the examined mediators, self-esteem was found partially mediated the relationship between family atmosphere and internet addiction. Moreover, we found that the pathway solely mediated by self-esteem had a greater influence than the other six pathways. Consequently, it can be inferred that an adverse family environment primarily diminishes one’s self-esteem rather than eliciting negative emotions to trigger internet addiction. Thus, implementing interventions that focus on enhancing self-esteem is likely to yield more positive results. The sociometric theory of self-esteem suggests that long-term exposure to an environment full of rejection, social exclusion and indifference reduces one’s self-esteem [67, 68]. Meanwhile, the cognitive-behavioral model of internet addiction proposed that low self-esteem constitutes a risk factor for internet addiction [69], which was validated by empirical findings in adolescents [8, 25]. The underlying mechanism of this mediation relationship may be that adolescents with low self-esteem due to inferior family atmosphere find it difficult to realize their values in real life but the virtual world, which may exacerbate their internet use and further increase the risk of internet addiction over time [70].

### The Mediating Role of Negative Emotions

The two examined negative emotions (i.e., anxiety and depression) mediated the association between family atmosphere and internet addiction in parallel and sequence. Previous studies showed that family atmospheres such as tension, lack of intimacy, and indifference led to anxiety and depression in adolescents [71, 72]. As a result, adolescents turned to internet use to escape reality and gain pleasure. This matched well with the self-medication model [73] and the compensatory theory of internet use [74], which assumes that internet use is an outlet for negative emotions caused by interior family atmospheres. Furthermore, the chain emotion-mediated pathway from anxiety to depression is consistent with previous models and findings [74, 75], implying anxiety may cause depression and later-on internet addiction. In addition, the relative strength of pathways with anxiety involved was larger than that of depression, emphasizing a more central role of anxiety than depression in the proposed mediation model.

### The Chain Mediation

It was also found that self-esteem, anxiety, and depression mediated the association between family atmosphere and internet addiction in sequence. Notably, the pathway of “family atmosphere—self-esteem—anxiety—internet addiction” was more important than the other three chain pathways. This is in line with the vulnerability model of emotion, indicating low self-esteem as a risk factor for anxiety [44]. Together with the salient effect of pathways with only self-esteem and anxiety as mediators found in the model, the present findings emphasized the critical roles of self-esteem and anxiety between inferior family atmosphere and internet addiction.

Altogether, the present findings confirmed and expanded the cognitive-behavioral model of pathological internet use [69] by showing that not only environmental (i.e., family atmosphere) and psychopathological factors (i.e., anxiety, depression), but negative perceptions (i.e., self-esteem) play a key role in making individuals seek success and escape from reality through the internet [76, 77].

### Implications

The present study has significant clinical implications. It suggests that addressing family atmosphere, self-esteem, anxiety, and depression may help adolescents reduce the risk of internet addiction. For instance, systemic family therapy (SFT [78, 79]) can improve family atmosphere and give warmth and mutual support, which may help promote adolescents’ self-esteem and further reduce the risk of internet addiction. As to negative emotions, cognitive behavior therapy (CBT [80, 81]) and solution-focused brief therapy (SFBT [82, 83]) were found effective in boosting emotions and arming people with effective emotion regulation skills.

### Limitations

This pioneering study used a relatively large sample to investigate the mediating role of self-esteem and negative emotions between family atmosphere and internet use in Chinese adolescents. Albeit the implications of our research findings, some limitations should be realized. Firstly, the sampling approach employed in this study guaranteed a representative sample of adolescents in Shanghai. However, it is imperative to recognize that these findings may not be universally applicable to all Chinese teenagers, much less to those worldwide. To improve the generalizability of these findings, future studies should explore more varied samples. Secondly, although the Hayes’s approach was eligible to test the proposed chain mediation model, the model’s goodness of fit cannot be established, necessitating the use of alternative software like AMOS to perform the model. Thirdly, although previous longitudinal studies generally supported that the mediators examined here are consequences rather than causes of inferior family atmosphere [12, 26, 35], their causal relationship with internet use remains relatively unclear. Specifically, anxiety and depression may interact with pathological internet use in a mutually self-enhancing way. Future longitudinal studies can help clarify their relationship beyond correlation. Lastly, Young’s internet addiction test (IAT-20 [55]) assesses internet use severity in general, while leaving the various purposes of use undistinguished. Of note, using the internet for some purposes (e.g., gaming, cyberbullying, sexting) may be more hazardous than others (e.g., searching literature, remote studying), and they are associated with different antecedent factors [84]. Hence, studies in the future can refine the present model by differentiating various internet use purposes.

### Conclusion

The present study demonstrated a strong negative relationship between adolescents’ family atmosphere and internet addiction, suggesting that an inferior family atmosphere posed a risk factor for internet addiction. More importantly, we found that this relationship was partially mediated by self-esteem and negative emotions (i.e., anxiety and depression) in parallel and sequence. And the pathway of family atmosphere—self-esteem—internet addiction played a more critical role than others. The present study revealed the pathogenesis of internet use from a comprehensive environment-personality-emotion perspective, which provides future intervention studies with important targeting factors.
